# Prevalence of Congenital Anomalies Among Pregnant Women at a Tertiary Care Center: A Cross-Sectional study

**DOI:** 10.7759/cureus.79444

**Published:** 2025-02-22

**Authors:** Dilasha Timilsina, Swati Gupta

**Affiliations:** 1 Department of Obstetrics and Gynaecology, Kathmandu Medical College, Kathmandu, NPL

**Keywords:** congenital anomalies, prenatal, prevalence, risk factors, screening

## Abstract

Introduction: Congenital anomalies are a major health problem and are responsible for a significant proportion of morbidity and mortality in newborns. The objective of this study was to find the prevalence and type of congenital anomalies along with the presence of factors responsible for their development among antenatal women and pregnancies delivered at Kathmandu Medical College.

Methods: This was a cross-sectional study conducted over a period of nine months in the Department of Obstetrics and Gynecology, Kathmandu Medical College. All congenital anomaly cases detected antenatally or at delivery were included in the study. Cases were analyzed to find out the prevalence, types of anomalies, and the risk factors involved.

Results: During the study period, 86 congenital anomalies were seen in 1565 cases of delivery and termination of pregnancy, showing a prevalence of 5.49%. Anomalies of the central nervous system (CNS) were the most common (n=29,33.72%) followed by the circulatory system (n=19, 22.10%). Congenital anomalies were mostly diagnosed by prenatal ultrasonography in 68 cases (79%). Among the cases of congenital anomalies, 56 (65%) had termination of pregnancy, 28 (32.60%) were live birth and two (2.40%) were stillborn. The maternal factor that was studied showed that congenital anomalies are frequently seen in the age group of 26-30 years (n=30,34.88%) and multiparous women (n=52, 60.47%).

Conclusions: The prevalence of congenital anomalies was high, and CNS anomalies were the most common anomaly observed in this study.

## Introduction

Congenital anomalies are structural, functional, behavioral, and metabolic disorders that occur during intrauterine life and can be detected prenatally, at birth, or later in infancy [1]. Congenital anomalies are the main cause of spontaneous abortion, stillbirth, and perinatal death as well as disability, which has a significant impact on individuals, families, and the healthcare system [2]. Congenital anomalies can involve a single organ or multiple organs and have been associated with various etiological factors. It is estimated that in around half of the congenital anomalies, etiology cannot be identified and in those where known etiology is present, 20-25% are multifactorial [3].

The average prevalence of congenital anomalies is 2.7%; of these 77% are live births and 17% are termination of pregnancy for fetal anomaly (TOPFA) [4]. According to the WHO, an estimated 240000 newborns die worldwide within 28 days due to congenital anomalies and nine out of 10 children born with serious congenital disorders are in low- and middle-income countries [5]. The prevalence is high in low socioeconomic countries like Nepal due to various factors such as unsupervised self-medication, poor antenatal care, nutritional deficiencies, exposure to teratogens, intrauterine infections, etc. [6]. Few studies have shown prevalence ranging from 0.93 to 3.5% in Nepal. However, the true magnitude is still unknown due to non-presentation to healthcare facilities, poor awareness, underreporting, etc. [6,7]. Most of these studies have either reported only congenital anomalies detected at birth or prenatal ultrasonography, but combined overall prevalence is seldom reported.

The objective of this study was to find the prevalence and pattern of congenital anomalies as well as the presence of factors responsible for the development of congenital anomalies among antenatal women and delivered babies at Kathmandu Medical College.

## Materials and methods

Study design and subjects

This is a hospital-based cross-sectional study conducted in the Department of Obstetrics and Gynecology, Kathmandu Medical College over a period of nine months from October 2023 to June 2024. All cases of delivery as well as termination of pregnancy during the study period were studied and congenital anomaly cases detected among them were included. Cases detected as congenital anomalies by antenatal ultrasound but found to be normal at delivery were excluded from the study. Antenatally detected cases were confirmed at delivery. Termination of pregnancy was done in cases with lethal anomalies after proper counseling. Ultrasound or X-ray of the baby was done in some cases as a part of neonatal screening in few cases. Informed consent was taken from all eligible mothers.

Congenital anomalies in this study were defined as “structural or functional defects that were detected by ultrasonography antenatally or identified post-delivery during neonatal screening, either clinically or through investigation modalities".

Sampling procedure

A consecutive convenience sampling technique was used to enroll the participants. All cases of delivery as well as termination of pregnancy during the study period were taken to find the prevalence of congenital anomalies.

Data collection technique

The source population was all antenatal mothers attending the obstetric OPD and labor room during the study period from October 2023 to June 2024. In all cases with congenital anomalies (delivery or termination of pregnancy), a thorough history regarding risk factors was taken and a clinical examination was performed. The pattern of congenital anomalies as well as the system involved was documented. The demographic profile of all mothers was recorded including the age, gravida, parity, antenatal checkup (ANC) visits, area of residence, gestational age at the time of detection of previous spontaneous abortions, folic acid intake, smoking or alcohol intake, family history of birth defects or birth defects in a previous pregnancy, history of exposure to drugs, radiation or history of fever in present pregnancy, etc. All required details were recorded in a structured proforma.

Statistical analysis

The data were collected and analyzed using IBM SPSS Statistics for Windows, Version 26 (Released 2019; IBM Corp., Armonk, New York, United States). The prevalence and pattern of congenital anomalies, mode of detection, and risk factors were recorded and expressed in frequencies and percentages.

Ethical consideration

Ethical approval was obtained from the Institutional Review Committee (IRC) of Kathmandu Medical College with reference number 29092023/24. The study manuscript was prepared according to the STROBE statement for cross-sectional studies [8]. Informed consent was taken from all participants.

## Results

A total of 1565 pregnancies were screened for congenital anomalies (1257 deliveries and 308 termination of pregnancy) of which 86 had congenital anomalies, resulting in a prevalence of 5.49%. The mean age of the patients with congenital anomalies in the fetus was 28.21 ± 4.90 years. The majority was seen in the age group of 26-30 years (n=30,34.88%) followed by the age group of 21-25 years (n=24, 27.90%). The lowest frequency of congenital anomalies was seen above 35 years of age (n=6, 7%). The incidence of congenital anomalies was higher in multiparous women (52 out of 86, 60.47%) and 31 (36%) cases were diagnosed to have anomalies after 30 weeks of gestation, whereas only thirteen cases (15.11%) were diagnosed before 16 weeks of gestation. Eighty (93%) patients were diagnosed on ANC visits. Residents of rural areas were more commonly associated with congenital anomalies (n=51, 59.30%). Only one patient had twin pregnancies while the rest were singleton. The maternal characteristics are shown in Table 1.

**Table 1:** Maternal parameters (N=86) ANC: Antenatal checkup

Variables	Frequency (n)	Percentage (%)
Maternal age (years)		
16-20	05	5.81
21-25	24	27.90
26-30	30	34.88
31-35	21	24.41
36-40	06	7.00
Parity		
Primi	34	39.53
Multi	52	60.47
Gestational age at diagnosis (weeks)		
<16	13	15.11
16-20	14	16.25
21-25	21	24.40
26-30	07	8.14
>30	31	36.10
Plurality		
Single	85	98.83
Multiple	01	1.17
ANC visits		
Yes	80	93
No	06	07
Residence		
Urban	35	40.70
Rural	51	59.30

Congenital anomalies were commonly diagnosed via antenatal ultrasound in 68 (79%) cases while the rest were diagnosed post delivery during neonatal screening. Out of 86 cases of congenital anomalies, 56 (65%) had TOPFA, 28 (32.60%) were live births and two (2.40%) were stillborn (Table 2).

**Table 2:** Mode of diagnosis and delivery (N=86) USG: Ultrasonography; TOPFA: Termination of pregnancy for fetal anomaly

Variables	Frequency (n)	Percentage (%)
Mode of Diagnosis		
USG	68	79
Neonatal Screening	18	21
Delivery		
TOPFA	56	65
Live birth	28	32.60
Still born	02	2.40

**Figure 1:**
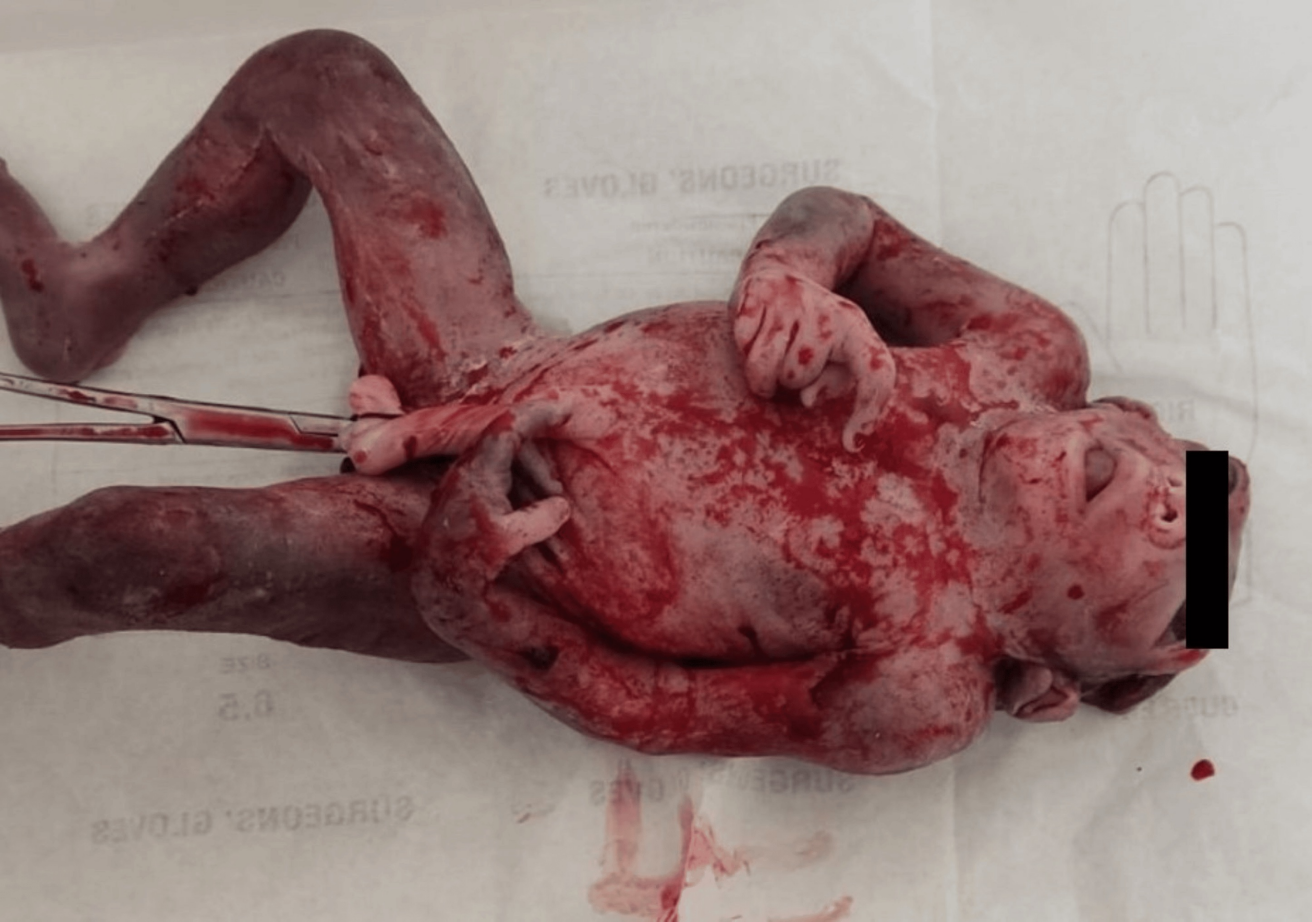
Congenital anomaly: anencephaly

**Figure 2:**
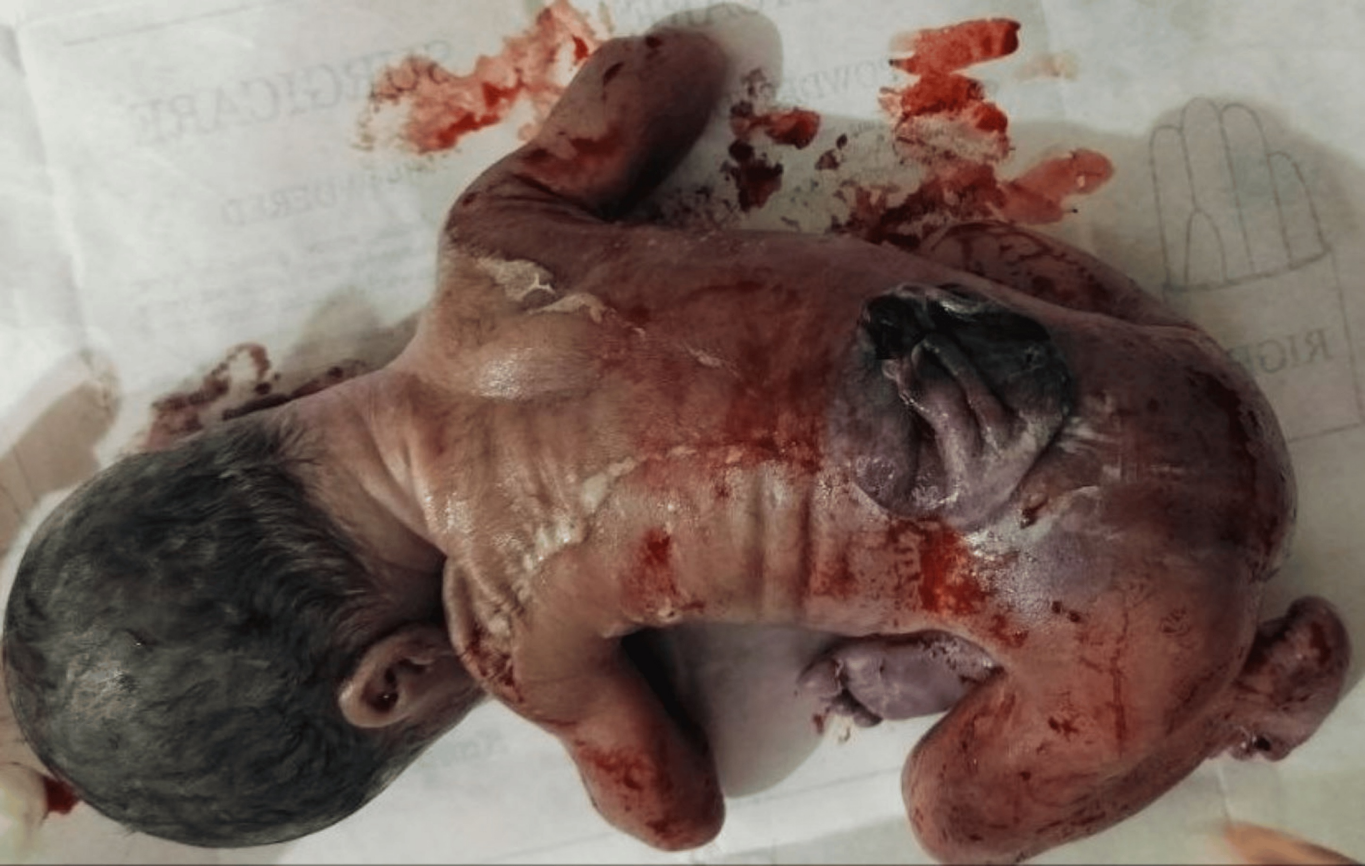
Congenital anomaly: meningomyelocele

**Figure 3:**
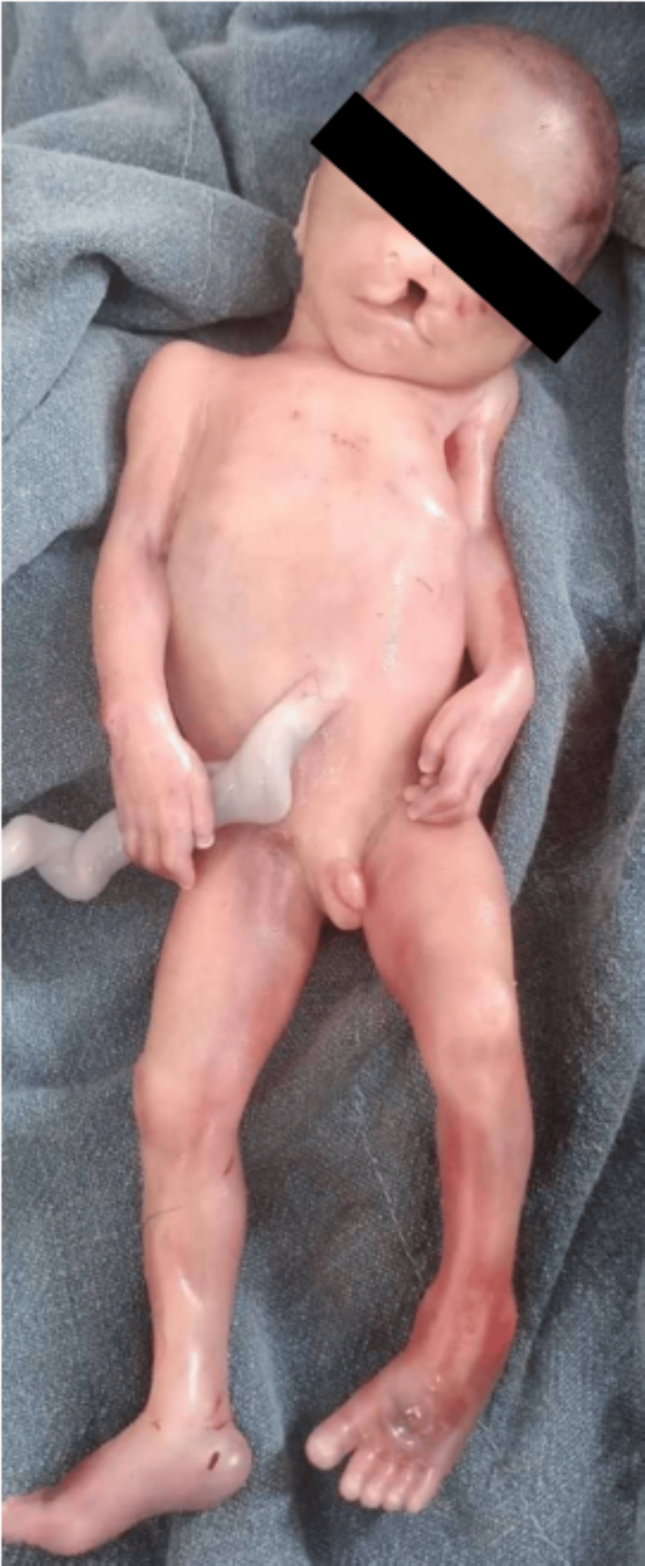
Congenital anomaly: cleft palate with a cleft lip

Congenital anomalies involving the central nervous system (CNS) (Figures 1, 2) were seen in 29 (33.72%) cases, of which anencephaly was the most frequently seen in six cases. The circulatory system was the second most commonly involved system, seen in 19 (22.10%) cases. Other systems involved were gastrointestinal (20.94%), musculoskeletal (9.3%), urinary (4.66%), cleft lip and palate (3.48%) (Figure 3), chromosomal abnormalities (3.48%), and respiratory (2.32%). The pattern of congenital anomalies seen in this study is shown in Table 3.

**Table 3:** Pattern of congenital anomalies (N=86) Classified According to the International Classification of Diseases (ICD 11) [9] CTEV: Congenital talipes equinovarus; CPAM: Congenital pulmonary airway malformation

S. No.	System (n)	Congenital anomaly	Frequency	Percentage (%)
1	Central nervous system (29)	Anencephaly	06	33.72
		Spina bifida	04	
		Hydrocephalous	03	
		Meningocele	03	
		Arnold Chiari Type 2	02	
		Dandy Walker malformation	02	
		Exencephaly	02	
		Sacrococcygeal teratoma	02	
		Ventriculomegaly	02	
		Aqueduct stenosis	01	
		Choroid plexus cyst	01	
		Meningomyelocele	01	
2	Circulatory system (19)	Ventricular septal defect (VSD)	05	22.10
		Hydrops fetalis	04	
		Coarctation of aorta	02	
		Hypoplastic left heart syndrome	02	
		Patent ductus arteriosus (PDA)	02	
		Tetralogy of Fallot (TOF)	02	
		Complete heart block	01	
		Supraventricular tachycardia	01	
3	Gastrointestinal (18)	Diaphragmatic hernia	04	20.94
		Gastroschisis	04	
		Anorectal atresia	03	
		Omphalocele	03	
		Tracheoesophageal fistula	02	
		Vitello intestinal duct	02	
4	Musculoskeletal (08)	Bilateral CTEV	04	9.30
		Duchenne muscular dystrophy	02	
		Skeletal dysplasia	02	
5	Urinary (04)	Dilated pelvicalyceal system	03	4.66
		Ectopic kidney	01	
6	Cleft lip and palate (03)	Cleft lip with cleft palate	02	3.48
		Cleft lip	01	
7	Chromosomal abnormalities (03)	Down syndrome	02	3.48
		Edward syndrome	01	
8	Respiratory (02)	CPAM	02	2.32

Various associated risk factors seen in this study were a history of previous spontaneous abortion (39.53%), lack of folic acid intake (5.81%), h/o drug intake (4.66%), oligohydramnios (3.48%), polyhydramnios (2.32%), intrauterine infections (2.32%), previous congenital anomaly (1.17%), twin pregnancy (1.17%), etc. Some patients had multiple risk factors combined. The associated risk factors noted in this study are shown in Table 4.

**Table 4:** Associated risk factors (N=86) PIH: Pregnancy-induced hypertension

SN	Risk factors	Frequency (n)	Percentage (%)
1	Previous spontaneous abortion	34	39.53
2	Lack of folic acid intake	05	5.81
3	H/o drug intake	04	4.66
4	Family history of congenital anomalies	03	3.48
5	Oligohydramnios	03	3.48
6	PIH	03	3.48
7	Intrauterine infections	02	2.32
8	Polyhydramnios	02	2.32
9	IgA nephropathy	01	1.17
10	Previous congenital anomaly	01	1.17
11	Twin pregnancy	01	1.17

## Discussion

Congenital anomalies are a major health problem and are responsible for a significant proportion of morbidity and mortality in newborns. They account for 3-5% of all pregnancies and 2-3% of all births [7]. According to the WHO, congenital anomalies are defined as structural or functional anomalies including metabolic disorders that are present at the time of birth [5]. The most common congenital anomalies encountered are heart defects, neural tube defects, Down syndrome, cleft lip and palate, club foot, hydrocephalus, anencephaly, etc. [7]. They can be detected during the antenatal period using ultrasonographic screening or post delivery during neonatal screening. The prevalence is high in low socioeconomic countries due to various factors like unsupervised self-medication, nutritional deficiencies, exposure to teratogens, intrauterine infections, poor antenatal care, etc. [6]. The prevalence and pattern of congenital anomalies may vary according to the geographical location representing an interaction of various factors like racial, ethnic, or sociocultural variations [10]. Several studies have been carried out to find the prevalence of congenital anomalies in different parts of Nepal with results ranging from 0.93 to 3.50% [6,7]. This study showed a prevalence of 5.49% which is higher compared to other studies from Nepal [6,7]. Most of these studies have either included only live birth defects or structural defects diagnosed by prenatal ultrasonography; however, our study included both birth defects and those detected prenatally. Prevalence data from other low-income countries like Nigeria showed a higher incidence of 6.30% compared to our study [1]. The frequency of birth defects in Nepal is low compared to other countries. Under-reporting of cases could be a major cause. Most congenital anomalies are reported from hospital data and anomalies in newborns delivered at home are missed. Nepal still has 57% of home deliveries [6]. In a community house household survey in Nepal, the prevalence of congenital anomalies was 52/10000 [7]. This demonstrates the burden of congenital anomalies among developing countries.

The maternal characteristics in this study are comparable to that in the literature. The mean age of the mother was 28.21± 4.90 years which is comparable to the study done by Anane-Fenin et al. in developing countries like ours [11]. Many studies have shown an association of increased maternal age with congenital anomalies, especially in mothers older than 35 years [12,13]. However, this was not found in our study as 93% of cases were below 35 years of age, comparable with few studies from Nepal and India [14,15]. This finding may be due to a smaller number of mothers who are older than 35 years in this study and less reported chromosomal anomalies which is often associated with advanced maternal age [16]. Another reason may be the early age of marriage and higher parity at an early age which is shown to increase the risk of congenital anomalies [3,12]. This study also showed a higher number of multiparous women (60.47%) having a congenital anomaly in the fetus.

An increasing number of congenital anomalies have been diagnosed by prenatal ultrasonography in the last decade. Advancements in USG machines, increased competence of radiologists, and increased access to USG have improved the rate of detection of congenital anomalies [15]. Seventy-nine percent of cases were detected via prenatal USG in this study, which is consistent with the findings of Kanhere et al. [3]. Increasing the detection rate prenatally also increases the TOPFA rate, as early detected major congenital anomalies are usually terminated. EUROCAT and global burden study showed a 17% TOPFA rate among congenital anomalies while the remaPAR study showed TOPFA of 26.80% [4,17,18]. This study has 65% cases of TOPFA, which is higher as compared to other studies. As our hospital is a tertiary referral center, many cases detected at prenatal screening or doubtful on initial assessment are referred for further evaluation, hence increasing the rate of TOPFA.

The pattern of congenital anomalies in this study showed the CNS as the most common (33.72%) system involved followed by the circulatory system, gastrointestinal tract, and musculoskeletal system, respectively. Similar findings were seen in studies conducted by others [3,14,19,20]. However, some studies showed the circulatory system as the most common while others showed either the gastrointestinal or musculoskeletal system as the common pattern [7,8]. Among CNS anomalies, anencephaly was the most frequently seen anomaly similar to other studies [3,14,20]. The difference in involved systems in various studies may be attributed to the methodology. For example, some studies enrolled only prenatal diagnosed cases while some involved only live births, and some focused only on neonates that were admitted [11].

Various risk factors associated with congenital anomalies were recorded in this study. Most of the cases had a history of spontaneous abortion in a previous pregnancy (39.53%) which is considered a risk factor as fetal chromosomal anomalies are associated with spontaneous abortion [7]. Lack of periconceptional use of folic acid was seen in 5.81% of cases and it is an established risk factor for congenital anomalies, especially neural tube defects. Hydramnios, intrauterine infection, and intake of teratogenic drugs are all known risk factors that were seen in this study as well as in a systematic review done in the African population [21]. Congenital anomalies in the family or previous pregnancy are an important risk factor and warrant strict screening during pregnancy for anomalies [7,19]. Many studies have shown that twin pregnancy is a risk factor for congenital anomalies; however, only one case of twin pregnancy in this study showed a congenital anomaly [3,7].

With the improving health care system in Nepal, an increasing number of pregnant women are subjected to prenatal USG and timely referral to tertiary care centers has increased the rate of detection of congenital anomalies and their management. However, geographical factors, lack of awareness among the public, and inadequate ANC visits hinder the evaluation as well as cause underreporting of congenital anomalies in Nepal. With high risk of recurrence of congenital anomalies, there are no well‑accepted preventive measures in countries like Nepal. It indicates that strong preventive measures for congenital anomalies are needed. Increasing awareness about maternal care during pregnancy, and educational programs on congenital malformations and their consequences need to be highlighted to decrease the incidence of congenital anomalies and their comorbidities.

There are a few limitations of this study. As it is a hospital-based study, it is unlikely to represent the national data. Our center is a tertiary referral center, and the prevalence of congenital anomalies may be higher compared to the general population. There was a small number of cases with risk factors; hence, an association of each factor could not be analyzed.

## Conclusions

The prevalence of congenital anomalies was high, and CNS anomalies were the most common anomalies observed in this study. Routine use of ultrasonography scans is an important measure for early detection of malformation, disability prevention, and decreasing perinatal morbidity and mortality. Early detection of fatal anomalies can avoid the continuation of pregnancy, thereby decreasing the economic burden and psychological trauma. Prenatal screening for early congenital anomaly detection should be an integral part of antenatal care.

## Appendices

Proforma

Prevalence of Congenital Anomalies Among Pregnant Women at Kathmandu Medical College

         Name:                                                 Address:                        IP no:

1)    General characteristics:

·       Age:

·       Parity: Nullipara/ Primipara/Multipara

·       Gravida:

·       Ethnicity: Hindu/Muslim/Christian/Buddhist/Others

·       ANC: Yes/No

·       No. of ANC visits:

·       Socioeconomic status

2)    Gestational age at diagnosis:

3)    Type of anomaly:

4)    Detection: USG/At delivery/Neonatal screening

5)    Fate: Delivered/Abortion/Stillbirth

6)    Risk factors:

·       Radiation exposure

·       Drugs

·       H/o folic acid

·       H/o DM

·       H/o consanguinity

·       Past h/o of anomalous baby

·       Family history of congenital anomaly

·       Congenital anomaly in the mother

·       Personal history of alcohol/ smoking

7)    USG findings:
